# Xanthogranulomatous Pyelonephritis in a Horseshoe Kidney: Effective Conservative Therapy in an Immunocompromised Patient

**DOI:** 10.7759/cureus.116028

**Published:** 2026-09-09

**Authors:** Mohnna Subahi, Ahmad M Albarakaty, Ahmed Almadaabgy

**Affiliations:** 1 Department of Urology, King Abdullah Medical City, Makkah, SAU; 2 Department of Urology, International Medical Center, Jeddah, SAU

**Keywords:** conservative, horseshoe kidney, pyelonephritis, xanthogranulomatous, xanthogranulomatous pyelonephritis

## Abstract

Xanthogranulomatous pyelonephritis (XGPN) is a rare, chronic granulomatous inflammation that leads to diffuse destruction of the renal parenchyma. This destructive process may be so profound as to infiltrate surrounding tissues and viscera. Although nephrectomy remains the most common option for managing XGPN in adults, we report a case of XGPN in a horseshoe kidney of a 27-year-old woman on active immunosuppressive therapy for inflammatory bowel disease, namely ulcerative colitis. This case was successfully treated conservatively with antimicrobials, with complete resolution of the renal mass and improvement in the general condition and inflammatory markers.

## Introduction

Xanthogranulomatous pyelonephritis (XGPN) is a chronic renal infection that leads to diffuse renal destruction [1]. In 1916, Schlagenhaufer first described this condition as a rare disease characterized by diffuse destruction of the normal renal parenchyma, which is subsequently replaced by distributed nodular and streaky granulomatous tissues. The replaced tissue is characterized by the composition of numerous lipid-laden macrophages (foam cells), which confer a distinctive yellowish-tan coloration.

Xanthogranulomatous pyelonephritis is a condition observed across all age groups, with reported cases ranging from 21 days to 90 years. However, it is most commonly seen in individuals over the age of 40 [2]. The clinical presentation of XGPN is nonspecific and variable, with the majority of patients presenting with symptoms such as flank or abdominal pain, fever, and the presence of a palpable mass. Rarely, vague gastrointestinal symptoms may occur as an atypical manifestation. Additionally, complications, including nephrocutaneous and nephrocolonic fistulas, as well as documented cases of psoas and paranephric abscesses, have been reported [3].

This condition can be classified into three distinct stages based on the extent of inflammation. In stage I, the inflammation is limited to the kidney, while in stage II, it involves both the kidney and the perirenal region. By stage III, the inflammatory process extends to include the kidney, perirenal, and diffuse retroperitoneal areas. Although total nephrectomy is generally considered the treatment of choice for all stages, segmental resection of the affected kidney may be a viable option in stage I and stage II cases [4]. This paper presents a rare case that was successfully managed with medical intervention alone, utilizing intravenous antibiotics without the need for invasive surgical procedures.

## Case presentation

A 27-year-old woman with a known diagnosis of ulcerative colitis, maintained on immunomodulators, presented to the emergency department during a disease flare with an oral ulcer and persistent fever of 10 days’ duration. She appeared cachectic, with a BMI of 17 kg/m² (underweight; normal range 18.5-24.9 kg/m²). The abdomen was soft and non-tender, and she reported no bothersome lower urinary tract symptoms. The complete blood count was unremarkable except for anemia, with a hemoglobin of 7.2 g/dL. The serum creatinine was 0.33 mg/dL, and the C-reactive protein (CRP) was markedly elevated at 17.7 mg/dL, while urine and blood cultures remained negative (Table 1).

**Table 1:** Laboratory investigations at presentation Reference ranges are those used at the treating institution. The hemoglobin was reduced, and the CRP was elevated, whereas the serum creatinine was low, consistent with the patient's reduced muscle mass. CRP: C-reactive protein

Parameter	Result	Reference range
Haemoglobin	7.2 g/dL	12.0-15.5 g/dL
Serum creatinine	0.33 mg/dL	0.5-1.1 mg/dL
CRP	17.7 mg/dL	< 0.5 mg/dL
White cell count	Within normal limits	4.0-11.0 × 10⁹/L
Urine culture	No growth	No growth
Blood culture	No growth	No growth

Abdominal CT demonstrated an average-sized horseshoe kidney without hydronephrosis. A heterogeneous, hypodense mass was seen at the lower pole of the left kidney, crossing the midline to involve part of the lower pole of the right kidney; it measured 5.5 × 2.0 × 4.0 cm and showed cystic change. Several enlarged lymph nodes were present in the left para-aortic region, the largest measuring approximately 1.2 cm (Figure 1). An isotopic dimercaptosuccinic acid (DMSA) scan revealed no renal scarring, with a split function of 47% in the left renal moiety. In the absence of calculi, obstruction, or positive cultures, the differential diagnosis of this mass included renal cell carcinoma, renal lymphoma, renal tuberculosis, and a pyogenic renal abscess; tissue diagnosis was therefore pursued.

**Figure 1:**
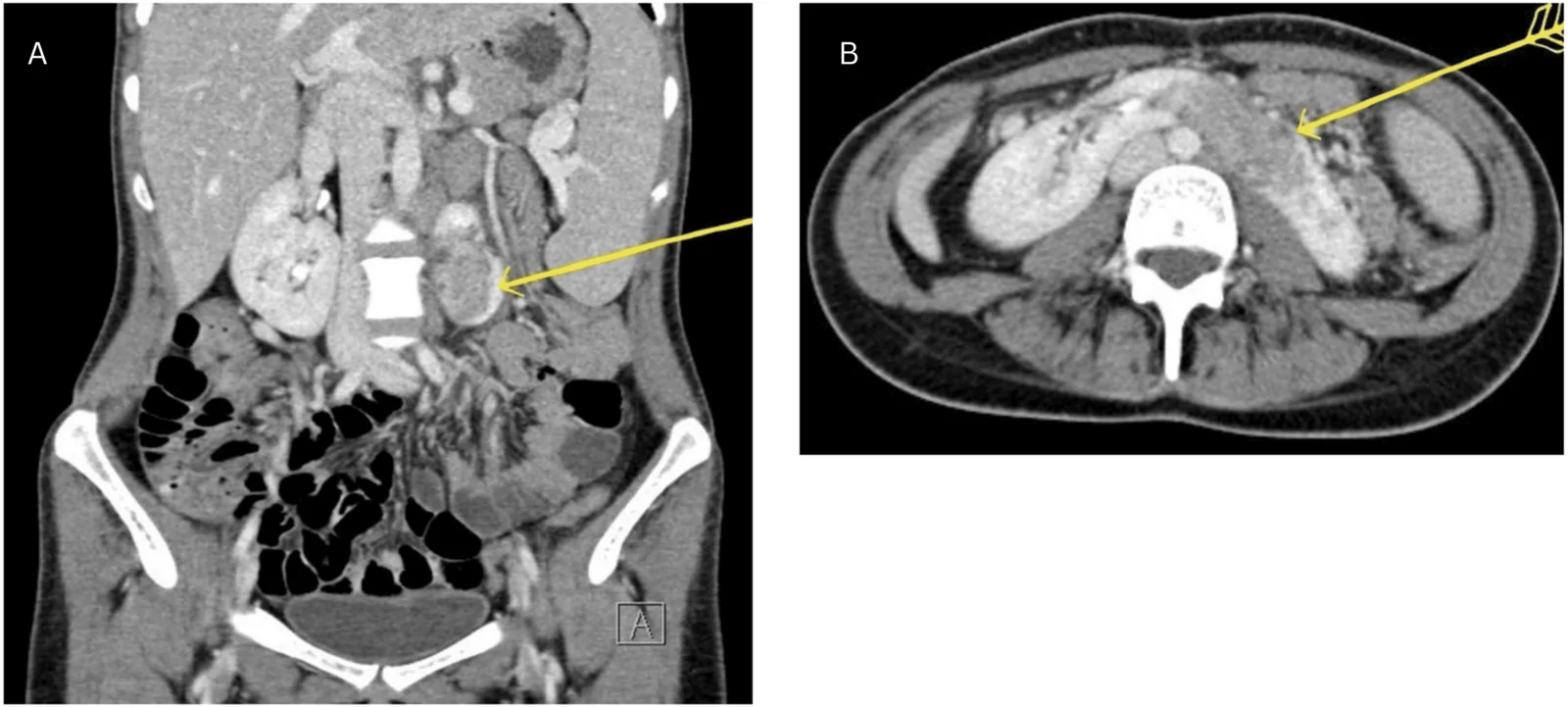
Abdominal CT Coronal (A) and axial (B) images show an average-sized horseshoe kidney without hydronephrosis. A heterogeneous, hypodense mass (yellow arrows) is present at the lower pole of the left kidney and crosses the midline to involve part of the lower pole of the right kidney.

A Tru-Cut needle biopsy was obtained. Histopathology showed linear cores of renal tissue containing some viable, intact glomeruli. The interstitium was moderately infiltrated by a mixed inflammatory population of lymphocytes, plasma cells, numerous histiocytes, eosinophils, and scattered neutrophils, together with a focal area of necrosis. The tubular epithelium was invaded by lymphocytes and neutrophils, and neutrophilic casts were seen within the tubules. These features were consistent with xanthogranulomatous pyelonephritis (Figure 2, panels A, B, and C). Mycobacterial and fungal infections were excluded using Ziehl-Neelsen and periodic acid-Schiff with diastase (PAS-D) stains. Immunohistochemistry demonstrated positive staining of the histiocytes for CD-68 and was negative for S-100, CD-117, and CD-1a (Figure 2, panel C).

**Figure 2:**
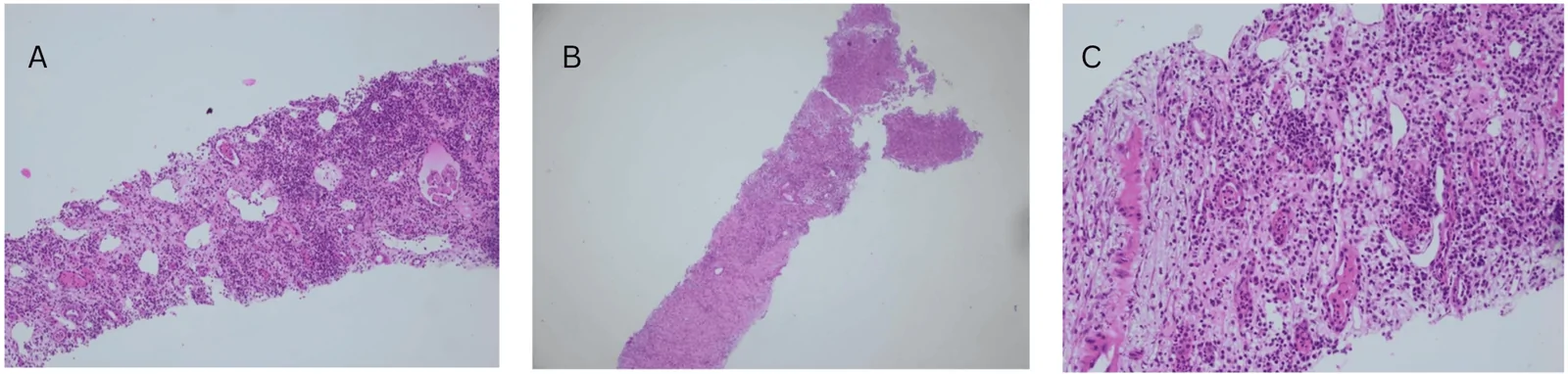
Histopathology of the Tru-Cut renal biopsy The sections show renal parenchyma with a mixed interstitial inflammatory infiltrate composed of lymphocytes, plasma cells, numerous histiocytes, eosinophils, and neutrophils, together with a focal area of necrosis and neutrophilic casts within the tubules; findings consistent with xanthogranulomatous pyelonephritis. Immunohistochemistry of the histiocytes was positive for cluster of differentiation 68 (CD-68).

Partial nephrectomy was reserved as a last resort because of the patient’s ongoing immunomodulatory treatment, the vascular complexity of the horseshoe kidney, and a thin-walled dilated colon that would have heightened the risk of bowel injury and reno-intestinal fistula [5]. The patient was therefore treated conservatively with antibiotics, with drainage held in reserve. With parenteral piperacillin/tazobactam (4.5 g every six hours), the patient remained clinically stable and afebrile for 10 days and showed steady clinical improvement. She was discharged on a 21-day course of oral antibiotics (cefuroxime 500 mg twice daily and nitrofurantoin 100 mg twice daily). A repeat abdominal CT one month later showed a reduction in the size of the renal mass with resolution of the central cystic/abscess areas, leaving residual linear parenchymal hypodensity that likely represented scarring. A further CT performed two months after completion of antibiotics confirmed complete resolution of the previously described mass (Figure 3).

**Figure 3:**
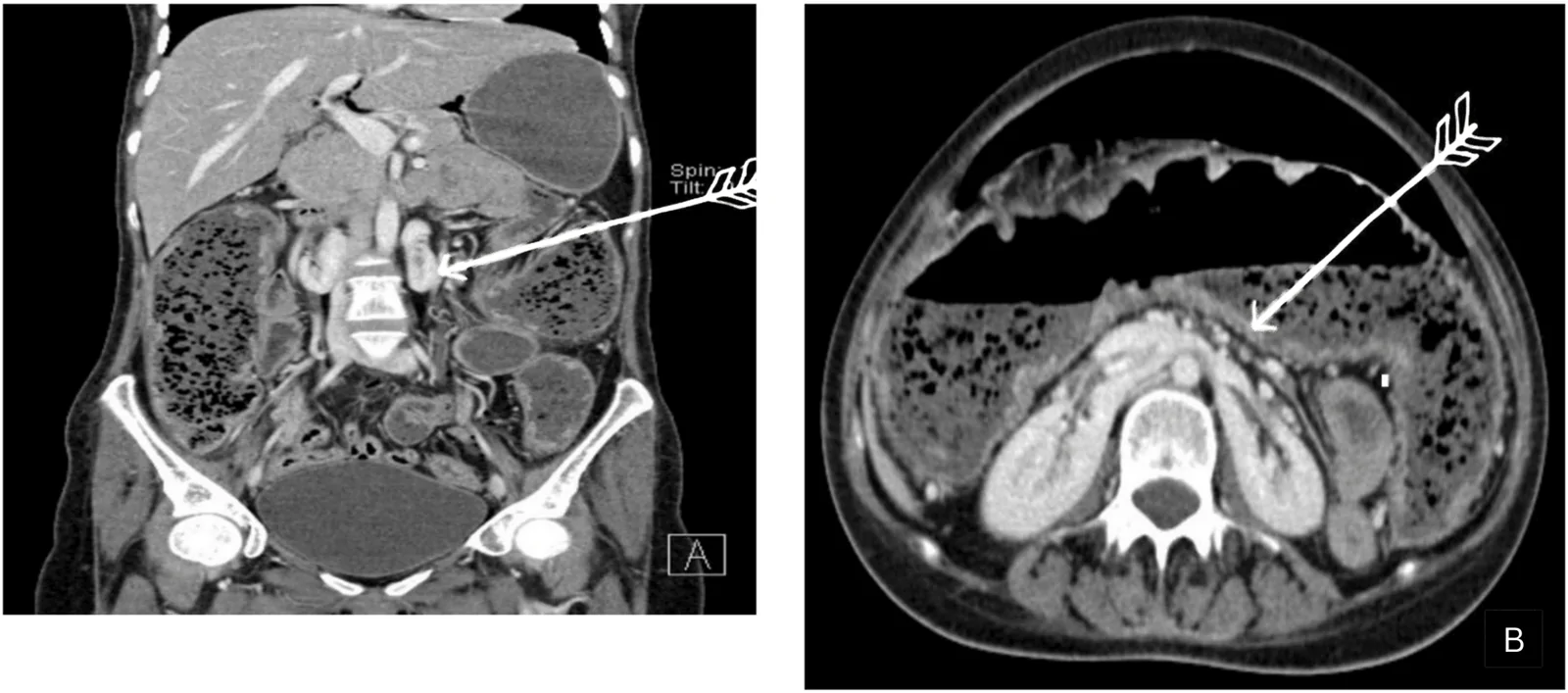
Follow-up abdominal CT Coronal (A) and axial (B) images show complete resolution of the previously described mass. The yellow arrows indicate the site of the resolved mass.

## Discussion

Xanthogranulomatous pyelonephritis represents an uncommon suppurative granulomatous reaction to chronic infection, most often in the setting of long-standing obstruction from a renal calculus [1]. *Escherichia coli *and *Proteus mirabilis* are the most frequently recovered organisms, and Gram-positive cocci such as *Staphylococcus aureus* and *Streptococcus faecalis* are also commonly identified; *Klebsiella* and *Pseudomonas* species have also been isolated from urine. Obstruction and urinary tract infection remain the principal predisposing factors for the disease [4,5].

Focal XGPN may closely imitate renal cell carcinoma (RCC) on imaging. Its heterogeneous, solid appearance on CT can be difficult to distinguish from a hypovascular RCC, particularly when neither urinary obstruction nor nephrolithiasis is present [6]. In our patient, blood and urine studies showed no evidence of active infection, and imaging revealed no obstruction. The differential diagnosis therefore includes malignant renal tumors and tuberculosis. Histologically, the presence of foamy, lipid-laden macrophages (xanthoma cells) accompanied by a dense infiltrate of plasma cells and histiocytes strongly supports the diagnosis [1].

Although antibiotic therapy alone has occasionally cured the disease, most often in children, nephrectomy remains the treatment used most frequently for XGPN. Image-guided interventional radiology, encompassing percutaneous aspiration, biopsy, drainage, stone extraction, and embolisation, contributes importantly to both diagnosis and management; nonetheless, these measures seldom obviate the eventual need for partial or total nephrectomy [7].

## Conclusions

Although nephrectomy remains the most common definitive treatment for XGPN in adults, this case demonstrates that complete clinical and radiological resolution can be achieved with antimicrobial therapy alone in appropriately selected patients. Features that favored a conservative approach in our patient included focal disease confined to the kidney, the absence of nephrolithiasis and urinary obstruction, preserved split renal function with no scarring on DMSA scanning, and prompt clinical improvement on empirical therapy. Percutaneous biopsy was essential in excluding malignancy, tuberculosis, and fungal infection before a non-operative course could be pursued with confidence. These considerations carry particular weight in a horseshoe kidney, where the vascular anatomy is complex and the functional cost of nephron loss is amplified, and in an immunosuppressed patient in whom surgical morbidity is increased. Conservative management should therefore be regarded as an individualized option requiring careful patient selection and close clinical and radiological surveillance, with drainage or surgery held in reserve, rather than as a general alternative to nephrectomy.
